# Examination of Early Childhood Temperament of Shyness and Social Avoidance and Associations With Cardiometabolic Health in Young Adulthood

**DOI:** 10.1001/jamanetworkopen.2021.44727

**Published:** 2022-01-27

**Authors:** Alva Tang, Nathan A. Fox, Natalie Slopen

**Affiliations:** 1 Department of Human Development and Quantitative Methodology, University of Maryland, College Park; 2 Department of Social and Behavioral Sciences, Harvard T.H. Chan School of Public Health, Harvard University, Boston, Massachusetts

## Abstract

**Importance:**

A child temperament characterized by shyness and avoidance of social interactions is associated with poor peer relationships and emotional problems, yet its long-term associations with adult cardiometabolic health are largely unknown.

**Objective:**

To examine whether a childhood temperament characterized by shyness and avoidance of social interactions is associated with poor cardiometabolic health.

**Design, Setting, and Participants:**

This cohort study included participants who were recruited at birth between April 1991 to December 1992 as part of a prospective longitudinal cohort, the Avon Longitudinal Study of Parents and Children. Follow-up assessments of health behaviors and cardiometabolic health extended into young adulthood (age 24 years). Data analysis was conducted between April and October 2021.

**Main Outcomes and Measures:**

Parent reports of temperament across ages 3 to 6 years were used to derive childhood temperament profiles in a longitudinal clustering analysis. Accelerometry measures of adolescent moderate to vigorous physical activity (MVPA) from ages 11 to 15 years and adult social occupation class were examined as mediators using path analyses. At age 24 years, 9 cardiometabolic outcomes were measured through anthropometrics and fasting blood samples: triglyceride levels, high-density lipoprotein (HDL) and low-density lipoprotein (LDL) cholesterol levels, glycated hemoglobin levels, insulin levels, systolic and diastolic blood pressure, body mass index, and C-reactive protein (CRP) levels.

**Results:**

Of the 9491 participants included in the analyses, 4908 (51.7%) were male, and 8668 of 9027 (96.0%) were White. Four childhood temperament profiles were identified: (1) introverted (2810 [29.6%]), (2) extraverted (2527 [26.6%]), (3) conflicted-shy (2335 [24.6%]), and (4) avoidant-shy (1819 [19.2%]). Lower childhood socioeconomic status was a precursor associated with the development of an avoidant-shy temperament (eg, introvert vs avoidant-shy: odds ratio, 1.13; 95% CI, 1.04-1.23). Path analyses showed that avoidant-shy children spent less time in MVPA in adolescence compared with all other temperament profiles (eg, introvert vs avoidant-shy: β = 0.10; *b* = 0.25; 95% CI, 0.14-0.35; *P* < .001), which in turn was associated with a cluster of cardiometabolic indices at age 24 years, including lower HDL cholesterol levels (β = 0.07; *b* = 0.95; 95% CI, 0.12-1.78; *P* = .03) and higher LDL cholesterol levels (β = −0.07; *b* = −1.69; 95% CI = −3.32 to −0.06; *P* = .04), insulin levels (β = −0.10; *b* = −0.57; 95% CI, −1.04 to −0.10; *P* = .02), diastolic blood pressure (β = −0.09; *b* = −0.59; 95% CI, −0.97 to −0.21; *P* = .002), and body mass index (β = −0.07; *b* = −0.32; 95% CI, −0.56 to −0.07; *P* = .01). Additionally, children classified as avoidant-shy attained lower social occupation classes at age 24 years, which was concurrently associated with higher BMI (β = 0.06; *b* = 0.21; 95% CI, 0.08-0.35; *P* = .002). Notably, these results were adjusted for a range of early developmental precursors and confounders, suggesting an independent association of temperament.

**Conclusions and Relevance:**

In this cohort study, less engagement in physical activity across adolescence seemed to be a developmental mechanism connecting an avoidant-shy childhood temperament and greater cardiometabolic risks over the life course. Future studies should examine the efficacy of physical activity and social skill programs that specially target the needs of different children to thereby reduce cardiometabolic risks.

## Key Points

**Question:**

Is a childhood temperament characterized by shyness and avoidance of social interactions associated with cardiometabolic health?

**Findings:**

In this cohort of 9491 children from the United Kingdom, children with an avoidant-shy temperament engaged in less physical activity in adolescence and attained lower social standing compared with children with other temperaments. These factors were associated with poor cardiometabolic health in adulthood.

**Meaning:**

These findings suggest that early temperamental differences among children are associated with long-term health behaviors and psychosocial development, which carry significant implications for cardiometabolic risks.

## Teaser

This cohort study examines whether a childhood temperament characterized by shyness and avoidance of social interactions is associated with poor cardiometabolic health.

## Introduction

Cardiovascular and metabolic diseases are the first and seventh leading causes of death across the world.^1^ It is now known from prospective observational studies of humans^2,3,4^ and animal models^5,6^ that many psychosocial risk factors and health behaviors affecting the course of cardiometabolic diseases emerge in early developmental periods. There is also recognition that group-level differences exist in the prevalence of early risk factors and disease incidents^7^; however, the downstream consequences of individual differences at the person level, particularly children’s temperament, are not well understood. Temperament refers to stable dispositions observed in early childhood that influence interpersonal interactions, psychosocial development, and health behaviors to shape cardiometabolic risk. Research identifying temperamental risks as early as possible can inform novel etiological models and prevention strategies that fit the needs of specific individuals.

**Figure 1.**
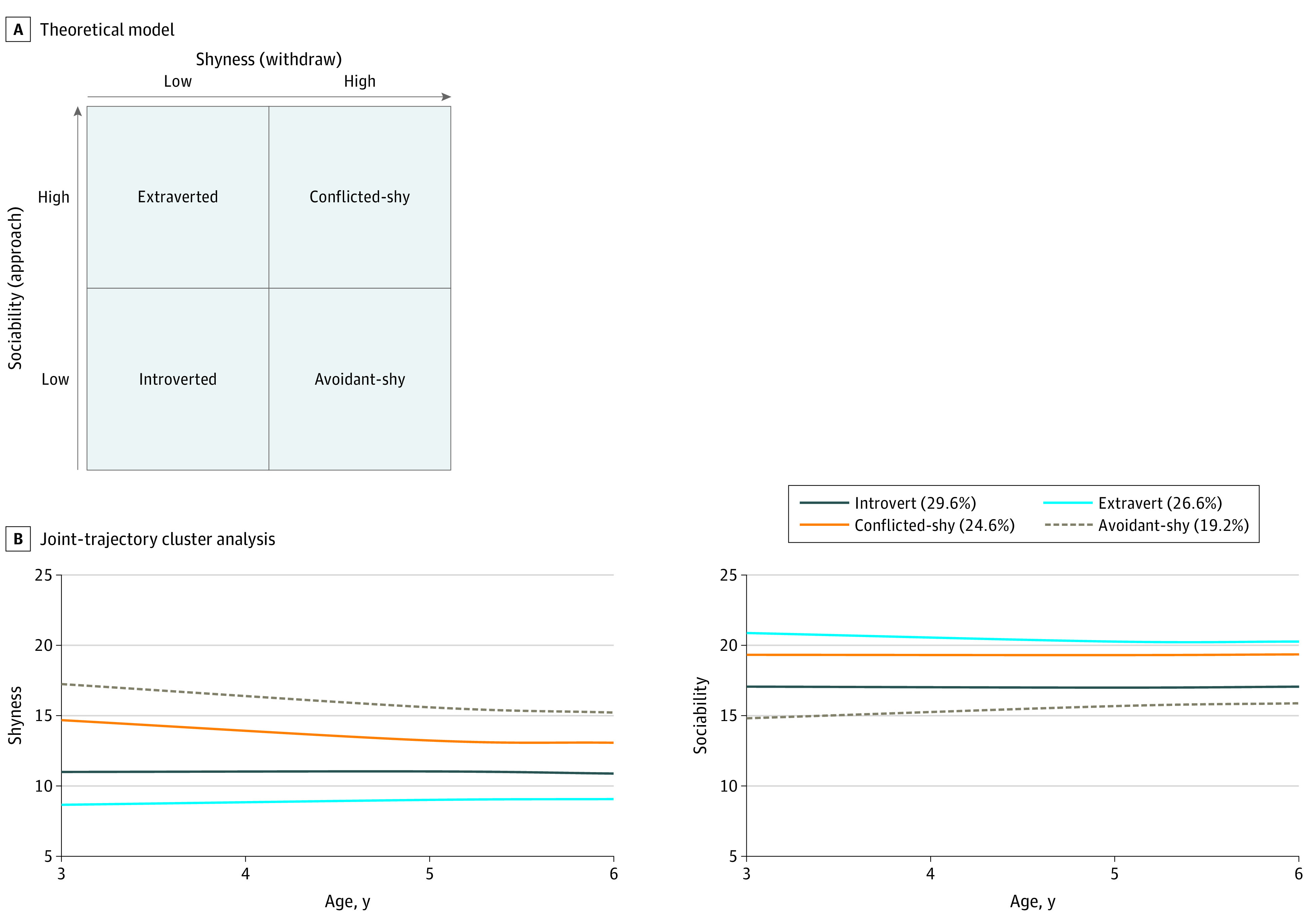
Theoretical Model Showing 4 Temperaments With Differences in Interpersonal Preferences Based on Shyness and Sociability and Empirical Results of the 4 Temperament Profiles Derived Through a Joint-Trajectory Cluster Analysis A, The theoretical model is adapted from Asendorpf.^19^ Overall, 9491 individuals were included in the analysis, including those with at least 2 data points of temperament. The introverted temperament is characterized by low shyness and low sociability; extraverted, low shyness and high sociability; conflicted-shy, high shyness and high sociability; avoidant-shy, high shyness and low sociability.

One temperament that is associated with poor psychosocial functioning across the lifespan is shyness. Shy children often express social withdrawal and lower social competence (ie, peer difficulties and fewer friends) in childhood.^8,9^ Such poor social relationships have been shown to persist into adulthood,^10,11,12^ which carry implications for adult health.^13,14^ Indeed, research in epidemiology has described associations between cardiometabolic risk and a distressed “type D” personality, reflecting shyness and social avoidance.^15,16,17,18^ However, this work is limited by cross-sectional or midlife prospective designs in adulthood, which muddle the direction of associations, as cardiometabolic diseases are already apparent and personality could result from the cardiometabolic conditions or other confounding conditions. In childhood, an avoidant-shy temperament can be identified through high levels of shyness (reflecting social inhibition) and low levels of sociability (reflecting amotivation to be with others)^19,20^ (Figure 1A). Objective observations in playgroups suggest that children with an avoidant-shy temperament are inhibited and actively avoid social situations by removing themselves from playgroups to relieve their anxiety, at the cost of missing out on opportunities to build social skills and friendships.^19,21,22^ Notably, this temperament is associated with negative outcomes, as children with an avoidant-shy temperament are rated as less likeable by peers and show higher depressive symptoms at follow-up in childhood compared with children with other temperaments.^22,23^ However, the long-term associations between child temperament and cardiometabolic health in adulthood, as well as the potential developmental pathways, remain unknown.

In this study, we used data from the Avon Longitudinal Study of Parents and Children (ALSPAC) to prospectively examine the long-term associations between child temperament and adult cardiometabolic health. We addressed 2 aims. First, we examined whether early social and biological factors before age 3 years (ie, childhood socioeconomic status [SES], birth weight accounting for gestational age, and maternal depression) are associated with an avoidant-shy temperament identified at ages 3 to 6 years. Based on prior studies, we hypothesized that childhood social disadvantage,^24^ maternal depressive symptoms,^25^ and low birth weight^26^ would be associated with an avoidant-shy temperament. Second, we identified developmental pathways through which child temperament could be indirectly associated with cardiometabolic risks. We tested 2 hypotheses. Following a health-behavior hypothesis, children with an avoidant-shy temperament might be socially disengaged, such that they may live sedentary lifestyles and be physically inactive, which could lead to elevated cardiometabolic risks. Following a differential-exposure hypothesis, children with an avoidant-shy temperament might attain lower social status in adulthood, which could contribute to elevated cardiometabolic risks.

## Methods

### Participants

**Figure 2.**
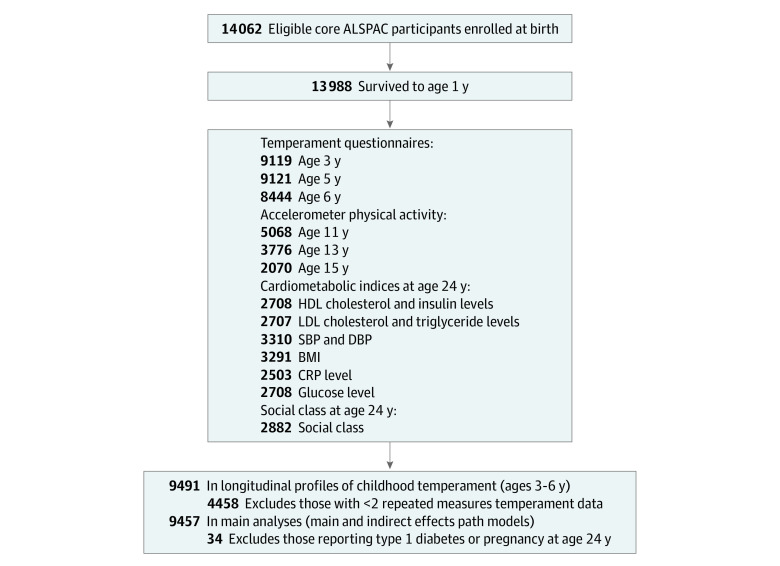
Participant Flowchart ALSPAC indicates Avon Longitudinal Study of Parents and Children; BMI, body mass index; CRP, C-reactive protein; DBP, diastolic blood pressure; HDL, high-density lipoprotein; LDL, low-density lipoprotein; and SBP, systolic blood pressure.

ALSPAC is a birth cohort study designed to investigate the developmental risk factors associated with physical and psychosocial health.^27,28,29^ Pregnant women from southwest England with expected delivery dates between April 1991 to December 1992 were recruited. The initial number of pregnancies enrolled was 14 541. Of these initial pregnancies, there were 14 676 fetuses, resulting in 14 062 live births and 13 988 children who were alive at 1 year of age. Figure 2 shows participant recruitment and data used in the current analyses. eAppendix 1 in the Supplement provides details on available data. Ethics approval for the study was obtained from the ALSPAC Law and Ethics Committee and local research ethics committees. Informed consent for the use of data collected via questionnaires and clinics was obtained from participants and parents. Children were invited to give assent where appropriate. Supporting documentation is available on the ALSPAC website.^30^ This study followed the Strengthening the Reporting of Observational Studies in Epidemiology (STROBE) guidelines.^31^

### Early Childhood Precursors, Aged 32 Weeks’ Gestation to 3 Years

#### Childhood SES

At 32 weeks’ gestation, parents reported their highest level of education (0, no high school diploma; 5, university degree) and occupations. Parents’ occupations were converted into standard occupation class, a proxy of social class, using a short-form of the National Statistics Socio-Economic Classification (NS-SEC).^32^ Social occupation class ranged from 1 to 5 (1, managerial/administrative positions and professional occupations; 5, semiroutine and routine occupations). At ages 2 and 3 years, mothers reported weekly family income on a 5-point scale (1, <£100; 5, >£400). Parental education, occupational class (reversed), and family weekly income were correlated (*r *ranged from 0.18 to 0.80; eTable 2 in the Supplement). These variables were standardized and averaged to provide a composite measure of early childhood SES.

#### Maternal Depressive Symptoms, Child Ages 2 to 3 Years

When participants were ages 2 and 3 years, mothers completed the 10-item Edinburgh Postnatal Depression Scale.^33^ Responses varied on a 4-point scale (0, no, not at all, or never; 3, yes, very often, or most of the time). Scores were correlated across time (*r* = 0.62). The average was calculated to provide a composite measure of maternal depressive symptoms.

#### Birth Weight *z* Score

Gestational age and birth weight were extracted from obstetric clinical records. These data were used to calculate sex-standardized birth weight *z* scores using the Fenton preterm growth chart.^34^

### Childhood Temperament, Ages 3 to 6 Years

At ages 3, 5, and 6 years, mothers completed the Emotionality Activity Sociability Temperament scale.^35^ The shyness and sociability subscales, each with 5 items, measured the child’s inhibition with unfamiliar people and preference to be in the company of others. Items measuring shyness and sociability include “child tends to be shy” and “child prefers playing with others than alone.” Responses varied on a 5-point scale (1, not at all like child; 5, exactly like child). In this sample, the 2 scales showed high internal consistency (α range for shyness, 0.79-0.83; α range for sociability, 0.63-0.68)^36^ and temporal stability (*r *range for shyness, 0.57-0.73; *r *range for sociability, 0.46-0.59) (eTable 3 in the Supplement). Shyness and sociability measures were used in a longitudinal cluster analysis to identify longitudinal temperament profiles (eAppendix 3 in the Supplement).

### Cardiometabolic Outcomes at Age 24 Years

Assessment of anthropometrics, blood pressure, and fasting blood samples were completed in the clinic. Nine cardiometabolic outcomes were measured: body mass index (BMI; calculated as weight in kilograms divided by height in meters squared), sitting systolic and diastolic blood pressure (SBP and DBP, respectively), triglyceride levels, high-density lipoprotein (HDL) and low-density lipoprotein (LDL) cholesterol levels, insulin level, glucose level, and C-reactive protein (CRP) level (eAppendix 2 in the Supplement). CRP, glucose, and insulin were log-transformed to reduce positive skewness.

### Potential Mechanisms

#### Adolescent Moderate to Vigorous Physical Activity

At ages 11, 13, and 15 years, children wore an accelerometer around the waist for a week during waking hours. The accelerometer detects acceleration and deceleration in a vertical plane due to movement frequency and intensity. A validated cutoff of moderate to vigorous physical activity (MVPA; ie, >3600 counts per minute [CPM]) in this sample was previously determined to be 4 times greater than the resting metabolic rate and approximates activity from brisk walking.^37^ Nonwear periods (periods with ≥10 minutes of continuous 0 CPM) and participants without the minimum valid hours and days of wear (<10 hours each day for 3 days) were excluded from analysis.^37^ Weekly MVPA was calculated and log-transformed to reduce positive skewness, then modeled as a latent variable (eAppendix 4 in the Supplement).

#### Social Status in Adulthood

At age 24 years, participants reported their occupation. This was converted into standard occupation classes using the NS-SEC.^32^

### Statistical Analysis

#### Preliminary Analysis

To identify distinct childhood temperament profiles, a nonparametric *k-*means longitudinal clustering method (ie, a simple form of machine learning) in the R package kml3d^38^ was used to detect joint trajectories of shyness and sociability across ages 3 to 6 years. A 4-cluster solution with relatively good fit and separation was identified (eTable 4 in the Supplement). To define a latent construct of adolescent physical activity measured by weekly MVPA at ages 11, 13, and 15 years, a confirmatory factor analysis was performed (eFigure 1 and eTable 5 in the Supplement). After defining these variables, they were added into path and structural equation models in the main analyses using the R package lavaan.^39^ Good model fit was determined by following conventional thresholds: comparative fit index, 0.95 and greater; root mean square error of approximation of 0.05 or less; and standardized root mean square residual of 0.08 or less.^40^

#### Main Analysis

To test the first aim—examining early precursors of temperament—a multinomial logistic regression model was performed in MPlus version 8 (Muthén & Muthén). Child temperament was entered as 3 dummy variables, with avoidant-shyness coded as the reference group (ie, introvert vs avoidant-shy, extravert vs avoidant-shy, and conflicted-shy vs avoidant-shy) and regressed on early precursors (ie, childhood SES, maternal depression, and birth weight *z* score). Unadjusted and adjusted models, which entered these precursors and the child’s sex and race, were performed. To test the second aim—whether child temperaments are indirectly associated with adult cardiometabolic outcomes via (1) adolescent physical activity and (2) attainment of adult social class—path models with indirect effects were performed (eFigure 2 in the Supplement). Statistical significance of the indirect effects was determined by 95% CIs calculated from 20 000 Monte Carlo simulations in the R package semTools.^41^ Unadjusted and adjusted models were performed.

#### Covariates

Adjusted models examining cardiometabolic outcomes accounted for 6 covariates that could be on the pathway to worse health: (1) child sex, (2) childhood SES, (3) maternal depressive symptoms, (4) child race (White vs other), (5) birth weight *z* scores, and (6) BMI at age 24 years. In models examining mechanisms, we additionally adjusted for the first 5 covariates, excluding BMI.

#### Missing Data and Solutions

Missing data were handled by full information maximum likelihood (FIML) estimation, which uses all available data in the analysis and is superior at reducing potential bias in parameter estimates compared with other methods (eg, imputation and listwise deletion).^42^ Figure 2 details participation and missing data. Of the 9491 participants with longitudinal child temperament profiles, those reporting type 1 diabetes or pregnancy at age 24 years (n = 34) were excluded from the main analyses, resulting in 9457 participants. Comparisons of the analyzed and unanalyzed groups indicated that the unanalyzed group came from more disadvantaged backgrounds (ie, belonged to racial minority groups, had lower childhood SES, or had mothers with greater depressive symptoms) and had lower birthweight *z* scores (eTable 1 in the Supplement). Participants with missing data on adolescent physical activity and outcomes at age 24 did not differ in the key variable, child temperament. Also, missingness in adolescent physical activity and adult cardiometabolic outcomes were unassociated with some demographic variables, including birth weight *z* scores; however, those with missing data were more likely to belong to a racial group other than White, to be male, to come from lower SES families, or to have mothers with greater depressive symptoms. These results suggest that the data are missing at random, which is a sufficient condition to use FIML estimation. We directly included these demographic variables in the models to provide more representative estimates. Of note, participants who had lower physical activity in adolescence were missing more cardiometabolic data at age 24 years. Accordingly, the associations between adolescent physical activity and adult cardiometabolic functioning are conservative estimates that cannot capture some physically inactive individuals.

## Results

### Precursors Associated With Different Child Temperaments

Of the 9491 participants included in the analyses, 4908 (51.7%) were male, and 8668 of 9027 with available data(96.0%) were White. Consistent with the shyness-sociability model, 4 joint trajectories across ages 3 to 6 years were identified (Figure 1B): (1) introverted group (2810 [29.6%]), characterized by low levels of both shyness and sociability; (2) extraverted group (2527 [26.6%]), characterized by low levels of shyness and high levels of sociability; (3) conflicted-shy group (2335 [24.6%]), characterized by high levels of both shyness and sociability; and (4) avoidant-shy group (1819 [19.2%]), characterized by high levels of shyness and low levels of sociability.

Characteristics of the 4 temperaments are shown in the Table. Significant early factors associated with these temperaments included childhood SES from birth to age 3 years and maternal depressive symptoms from ages 2 to 3 years in unadjusted and adjusted models (eTable 6 in the Supplement). Children from lower SES families were more likely to be in the avoidant-shy group compared with all 3 other temperaments (adjusted estimates: introvert vs avoidant-shy: odds ratio [OR], 1.13; 95% CI, 1.04-1.23; extravert vs avoidant-shy: OR, 1.25; 95% CI, 1.14-1.36; conflicted-shy vs avoidant-shy: OR, 1.13; 95% CI, 1.04-1.23). Compared with mothers of children with an avoidant-shy temperament, mothers of children with an introverted temperament reported fewer depressive symptoms (adjusted estimate: OR, 0.98; 95% CI, 0.96-0.99). Birth weight *z* score was not associated with temperaments.

**Table.** Characteristics of the 4 Childhood Temperaments

Characteristic	Participants, No. (%)^a^			
	Introvert	Extravert	Conflicted-shy	Avoidant-shy
No. (%)	2810 (29.6)	2527 (26.6)	2335 (24.6)	1819 (19.2)
Characteristics of trajectories				
Female sex	1261 (44.9)	1239 (49.0)	1261 (54.0)	822 (45.2)
Male sex	1549 (55.1)	1288 (51.0)	1074 (46.0)	997 (54.8)
White race	2566 (96.2)	2295 (95.7)	2145 (96.4)	1662 (95.8)
Maternal depressive symptoms, mean (SD)	5.62 (4.41)	6.07 (4.60)	6.06 (4.41)	6.17 (4.46)
Childhood SES factor score, mean (SD)	0.00 (0.71)	0.04 (0.71)	−0.01 (0.72)	−0.07 (0.72)
Birthweight *z* score, mean (SD)	−0.13 (0.92)	−0.20 (0.94)	−0.18 (0.95)	−0.17 (0.95)
Standard occupation class age 24 y, mean (SD)^b^	2.34 (1.60)	2.23 (1.56)	2.29 (1.60)	2.55 (1.69)
Cardiometabolic functioning age 24, median (IQR)				
CRP, mg/dL	0.077 (0.040- 0.203)	0.095 (0.040-0.255)	0.080 (0.036-0.197)	0.080 (0.037-0.204)
BMI	23.76 (21.62-26.81)	24.19 (21.78-27.39)	23.39 (21.28-26.93)	23.00 (21.14-26.01)
Triglycerides, mg/dL	75.28 (58.90-102.30)	74.40 (57.57-104.07)	72.63 (56.68-98.09)	74.40 (57.13-101.86)
LDL cholesterol, mg/dL	92.74 (74.85-111.24)	92.03 (75.01-112.32)	89.82 (71.44-112.24)	89.66 (73.14-112.35)
HDL cholesterol, mg/dL	57.62 (47.95-67.67)	59.17 (48.72-70.38)	59.55 (48.72-70.38)	58.01 (48.72-69.61)
Glucose, mg/dL	94.41 (89.01-101.08)	94.95 (89.37-100.54)	94.14 (89.37-101.44)	94.23 (89.01-100.18)
Insulin, μU/mL	7.38 (5.33-10.75)	7.62 (5.32-10.99)	7.43 (5.19-10.67)	7.04 (5.33-10.06)
Blood pressure, mm Hg				
Systolic	116.00 (108.00-123.33)	115.00 (108.00-123.58)	114.67 (107.00-123.00)	115.00 (107.83-123.00)
Diastolic	66.00 (60.67-71.67)	66.67 (61.83-71.67)	66.00 (61.00-72.00)	66.50 (61.67-71.50)

Abbreviations: BMI, body mass index (calculated as weight in kilograms divided by height in meters squared); CRP, C-reactive protein; DBP, diastolic blood pressure; HDL, high-density lipoprotein; LDL, low-density lipoprotein; SBP, systolic blood pressure; SES, socioeconomic status.

SI conversion factors: To convert CRP to milligrams per liter, multiply by 10; glucose to millimoles per liter, multiply by 0.0555; HDL and LDL cholesterol to millimoles per liter, multiply by 0.0259; and triglycerides to millimoles per liter, multiply by 0.0113.

^a^ The number of participants with data varied for each measure: 9491 for temperament profiles, sex, and maternal depressive symptoms; 9027, child race; 9432, childhood SES; 9384, birth weight *z* score; 2708, HDL cholesterol, insulin, and glucose levels at age 24 years; 2708, LDL cholesterol and triglyceride levels at age 24 years; 3310, SBP and DBP; 3291, BMI; 2503, CRP levels, and 2882, social occupation class.

^b^ Higher values of standard occupation class represent lower occupation classes.

Associations between child temperaments and outcomes at age 24 years are shown in eTable 7 in the Supplement. The children with an avoidant-shy temperament had lower BMI at age 24 years compared with the 3 other temperaments in both unadjusted and adjusted models (adjusted estimates: introvert vs avoidant-shy: β = 0.08; 95% CI, 0.03-0.12; extravert vs avoidant-shy: β = 0.11; 95% CI, 0.07 to 0.16; conflicted-shy vs avoidant-shy: β = 0.06; 95% CI, 0.01-0.10). Despite having lower BMI, children with an avoidant-shy temperament had higher levels of CRP at age 24 years compared with other shy temperaments who could appear socially withdrawn (ie, those with introverted and conflicted-shy temperaments). However, CRP levels were not different between the avoidant-shy and extraverted groups.

### Developmental Pathways Between Child Temperaments and Adult Cardiometabolic Outcomes

#### Physical Activity Across Adolescence

**Figure 3.**
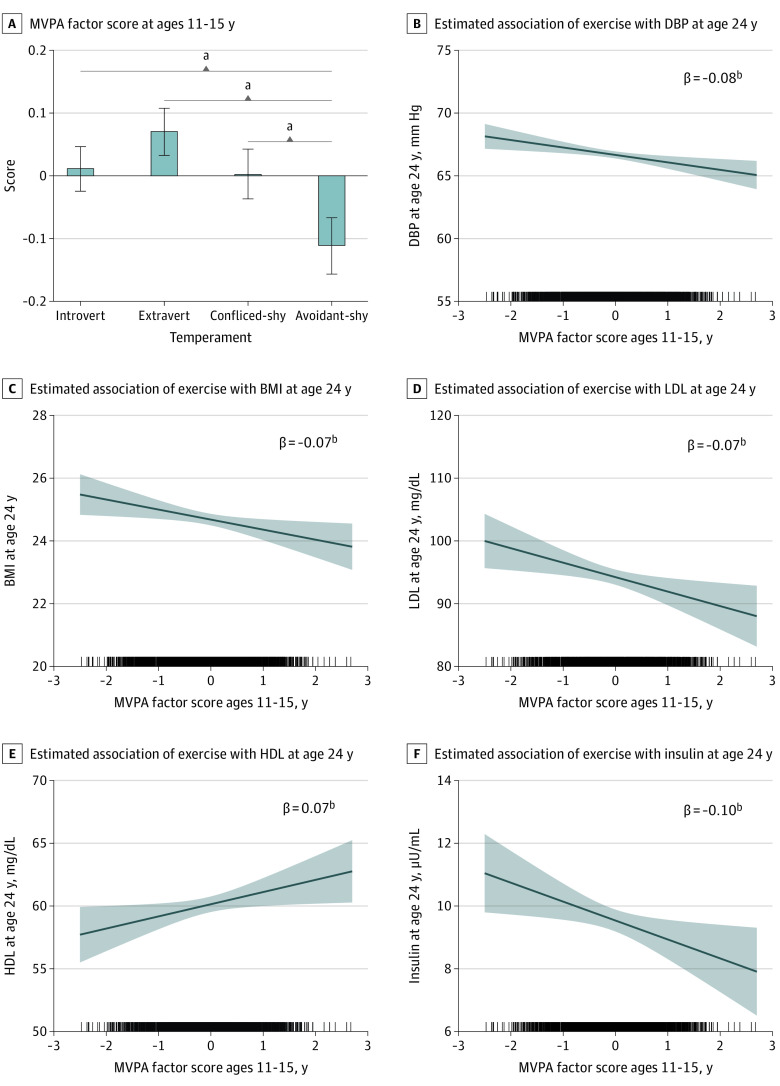
Association of Moderate to Vigorous Physical Activity (MVPA) in Adolescence With Cardiometabolic Outcomes in Young Adulthood and MVPA by Childhood Temperament B-F, Shaded areas indicate 95% CIs. All analyses adjusted for sex, child race, childhood socioeconomic status, maternal depressive symptoms, birth weight, and body mass index (BMI) at age 24 years. The analysis for BMI does not include itself as a covariate. DBP indicates diastolic blood pressure; HDL, high-density lipoprotein; and LDL, low-density lipoprotein. To convert HDL and LDL cholesterol to millimoles per liter, multiply by 0.0259. ^a^*P* < .001. ^b^*P* < .05.

**Figure 4.**
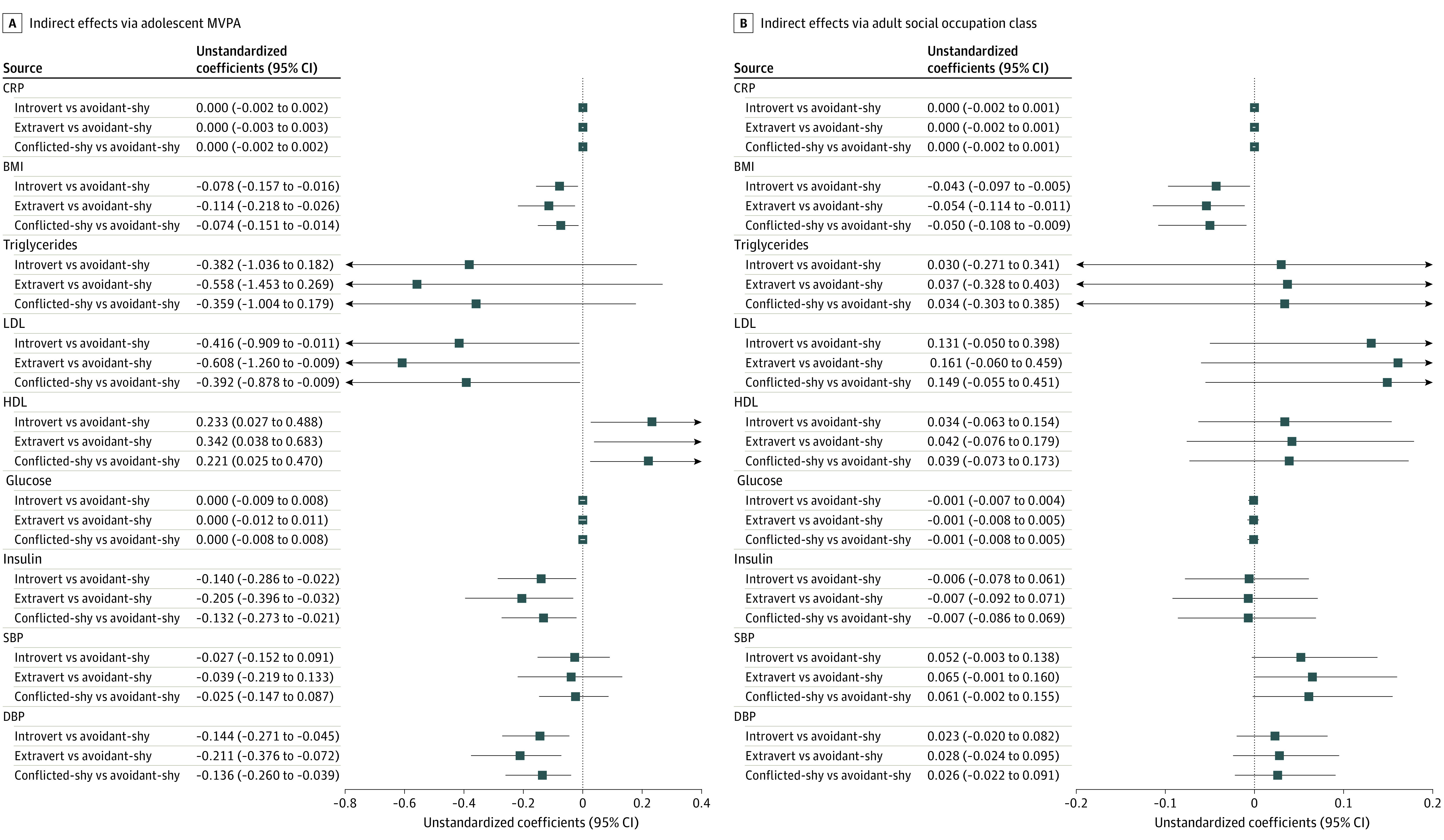
Unstandardized Indirect Associations Between Child Temperaments and Cardiometabolic Outcomes via Moderate to Vigorous Physical Activity (MVPA) in Adolescence and via Social Occupation Class in Young Adulthood Forest plots show estimates of indirect effects from path models and 95% CIs derived from Monte Carlo simulations. Estimates with 95% CIs that do not cross the horizontal line at zero indicate that the indirect associations are significantly different from zero. Higher values of standard occupation class in adulthood represent lower occupation classes. BMI indicates body mass index; CRP, C-reactive protein; DBP, diastolic blood pressure; HDL, high-density lipoprotein; LDL, low-density lipoprotein; and SBP, systolic blood pressure.

Figure 3 shows the MVPA factor scores in adolescence by child temperaments. Children with an avoidant-shy temperament spent less time in MVPA across adolescence compared with all 3 other temperaments (introvert vs avoidant-shy: β = 0.10;* b* = 0.25; 95% CI, 0.14-0.35; *P* < .001; extravert vs avoidant-shy: β = 0.14; *b* = 0.36; 95% CI, 0.25-0.47; *P* < .001; conflicted-shy vs avoidant-shy: β = 0.09; *b* = 0.23; 95% CI = 0.12-0.34; *P* < .001). In turn, less MVPA in adolescence was associated with higher levels of LDL cholesterol (β = −0.07; *b* = −1.69; 95% CI = −3.32 to −0.06; *P* = .04), insulin (β = −0.10; *b* = −0.57; 95% CI, −1.04 to −0.10; *P* = .02), BMI (β = −0.07; *b* = −0.32; 95% CI, −0.56 to −0.07; *P* = .01), DBP (β = −0.09; *b* = −0.59; 95% CI, −0.97 to −0.21; *P* = .002) and lower HDL cholesterol levels (β = 0.07; *b* = 0.95; 95% CI, 0.12-1.78; *P* = .03) at age 24 years (Figure 4; eTables 8 and 9 in the Supplement). Follow-up analyses indicated that these indirect associations between an avoidant-shy temperament and adult cardiometabolic indices via lower MVPA in adolescence were significantly different from zero (Figure 4). However, MVPA in adolescence was not associated with CRP, glucose, and triglycerides levels or SBP.

#### Social Status in Adulthood

Children with an avoidant-shy temperament attained lower social occupation classes compared with all 3 other child temperaments (introvert vs avoidant-shy: β = −0.06; *b* = −0.21; 95% CI = −0.38 to −0.04; *P* = .02; extravert vs avoidant-shy: β = −0.08; *b* = −0.26; 95% CI, −0.43 to −0.08; *P* = .004; conflicted-shy vs avoidant-shy: β = −0.07; *b* = −0.24; 95% CI, −0.41 to −0.07, *P* = .007). Indirect path models showed no significant associations between social occupation class at age 24 years and cardiometabolic measures, except for BMI (β = 0.06; *b* = 0.21; 95% CI, 0.08-0.35; *P* = .002) (eTable 10 and 11 in the Supplement). Follow-up analyses indicated that the indirect effect of an avoidant-shy temperament on BMI via adult social class was significantly different from zero (Figure 4).

## Discussion

Using a population-based prospective study, our results suggest 3 novel insights regarding temperamental etiologies and their downstream associations with developmental pathways underlying cardiometabolic health in young adulthood. First, children from socially disadvantaged families were disproportionately likely to be classified with an avoidant-shy temperament. This finding highlights the early socioeconomic environmental association with temperament development, which is largely overlooked by child temperament studies that rely on relatively smaller samples from middle-to-high socioeconomic backgrounds.^43,44^ This result converges with an Australian cohort reporting that children from families with disadvantaged SES were less sociable and more emotionally reactive.^24^ Children with an avoidant-shy temperament also grew up ranking lower in social occupation class, which suggests a novel temperament developmental pathway through which early social disadvantage could intergenerationally sustain less upward social mobility. This chain of associations in development is likely probabilistic rather than deterministic. An avoidant-shy temperament likely diminishes the social capital, including the quality and quantity of social relationships and interpersonal skills, that the child will accumulate and draw on during challenges throughout the life course.

Second, differences in child temperaments generally were not directly associated with adult cardiometabolic functioning, except for inflammation, which promotes arterial plaque and development of cardiovascular diseases.^45^ Elevated CRP levels at age 24 years were observed in individuals with an avoidant-shy childhood temperament compared with others who could appear socially withdrawn (ie, children with introverted and conflicted-shy temperaments). This association was independent of a range of well-established precursors and confounders associated with poor health. Additionally, this result was present despite the individuals with an avoidant-shy childhood temperament having lower BMI by age 24 years compared with the 3 other temperaments. These findings extend prior studies reporting associations between a type D personality and increased cardiovascular risks in midadulthood.^15,16,17,18^ A related line of work also suggests that childhood social isolation is prospectively associated with cardiovascular disease risks^4,13,46^; however, children could be socially isolated for various external (eg, peer exclusion and bullying^3^) or internal (eg, temperament) reasons. Here, our evidence suggests that a child temperament, reflecting a combination of social inhibition and amotivation for social interactions, can also shape inflammatory tendencies by young adulthood.

Third, in examining developmental pathways, we found that children with an avoidant-shy temperament engaged in less MVPA in adolescence compared with the 3 other temperaments. In turn, less MVPA was associated with a cluster of cardiometabolic outcomes, including higher insulin, DBP, BMI, and LDL cholesterol levels as well as lower HDL cholesterol levels by age 24 years. These findings are consistent with a health-behavior hypothesis^47^ and suggest that individuals with an avoidant-shy childhood temperament may be at increased risk of later cardiometabolic diseases because they are less physically active. Notably, physical activity is a modifiable mechanism, with interventions among youths demonstrating increases in MVPA can effectively reduce cardiometabolic risks.^48,49^ Future prospective randomized clinical trials are needed to provide evidence on the efficacy of various types of intervention that aim to improve the health of children with avoidant-shy temperaments, including physical activity alone vs physical activity in an organized group with peer interactions or in combination with programs that focus on improving social competence. Furthermore, there was partial support for a stress-exposure hypothesis,^48,49^ as individuals with an avoidant-shy childhood temperament attained lower social occupation classes at age 24 years compared with the other temperaments. In turn, lower social class was concurrently associated with elevated BMI at age 24 years. As this cohort ages into midadulthood, it will be important to examine whether additional associations emerge.

### Strengths and Limitations

Strengths of this study include the application of (1) a life-course perspective in a prospective and representative cohort; (2) a data-driven statistical technique to verify the distinct temperaments; and (3) objective measures of cardiometabolic health and physical activity.

However, there were several limitations. First, temperament measures were subjective parent reports, which could be biased by the parents’ perception. Therefore, future studies are needed to examine more objective measures, including using blinded observers to code children’s shy and sociable behaviors during interactions with unfamiliar peers^21,43^ and multi-informant reports from parents, teachers, and peers. Second, missing data were more common among children who belonged to racial minority groups, those from families with lower SES, and those whose mothers had higher depressive symptoms. While our models estimated for these variables to minimize bias, future research should increase recruitment efforts to retain a more representative and diverse sample. Third, individuals who were less physically active as adolescents were missing more adult cardiometabolic data. This missing pattern potentially leads to biased estimations of the association of adolescent physical activity with cardiometabolic indices, although in a conservative direction, as we could not capture a portion of physically inactive adolescents who would be expected to have poor cardiometabolic health at age 24 years. Fourth, there remains residual confounding from unmeasured variables, such as early childhood intelligence. Fifth, to extend the generalizability of results, future studies from other countries and cultures are needed.

## Conclusions

In this study, an avoidant-shy temperament in childhood was associated with core mechanisms underlying cardiometabolic health across the life course. Preventions designed to promote health behaviors should target specific children, who are shy and socially avoidant and require additional motivation to engage in physical and social activities. Future longitudinal research integrating child temperament could advance the understanding of individual differences in response to social environments and the design of prevention-oriented interventions.

## Notes

Supplement. **eAppendix 1. **Description of Sample **eTable 1.** Details of Data Included and Demographic Characteristics of Participants Included in Main Analysis Compared With Those Who Were Not Due to Missing Data, Pregnancy, or Type 1 Diabetes **eAppendix 2. **Assessment of Cardiometabolic Outcomes in Adulthood (Age 24 Years) **eTable 2.** Bivariate Correlations Among Measures of Childhood Socioeconomic Status **eAppendix 3. **Longitudinal Temperament Profiles Using Repeated Measures of Shyness and Sociability **eTable 3.** Bivariate Correlations Among Measures Of Shyness and Sociability Across Ages 3 to 5 Years **eTable 4. **Fit and Quality of Partition Statistics From Longitudinal Clustering Analysis **eAppendix 4. **Adolescent Moderate to Vigorous Physical Activity (MVPA) **eFigure 1.** Conceptual Diagram of the Measurement Model of Adolescent Physical Activity **eTable 5.** Factor Loadings and Fit Indices for the Confirmatory Factor Analysis of Adolescent Physical Activity **eFigure 2.** Conceptual Figure of the Path Analyses and Structural Equation Models Examining Indirect Effects of Childhood Temperaments on Cardiometabolic Outcomes via Social Occupation Class in Adulthood and Physical Activity in Adolescence **eTable 6.** Unadjusted and Adjusted Associations Between Early Social and Biological Factors and Child Temperament **eTable 7.** Unadjusted and Adjusted Associations Between Child Temperament, Adolescent MVPA, and Young Adult Social Occupation Class and Cardiometabolic Indices at Age 24 Years **eTable 8.** Results From Models Examining the Indirect Effects of Child Temperament on CRP Levels, BMI, Triglyceride Levels, and HDL and LDL Cholesterol Levels at Age 24 Years, via Adolescent Physical Activity **eTable 9.** Results From Models Examining the Indirect Effects of Child Temperament on Glucose Levels, Insulin Levels, SBP, and DBP at Age 24 Years, via Adolescent Physical Activity **eTable 10.** Results From Models Examining the Indirect Effects of Child Temperament on CRP Levels, BMI, Triglyceride Levels, and HDL and LDL Cholesterol Levels at Age 24 Years, via Adult Social Occupation Class **eTable 11.** Results From Models Examining the Indirect Effects of Child Temperament on Glucose Levels, Insulin Levels, SBP, and DBP at Age 24, via Adult Social Occupation Class
eReferences.
