# Supplementary material for: Examination of Early Childhood Temperament of Shyness and Social Avoidance and Associations With Cardiometabolic Health in Young Adulthood
Source: JAMA Netw Open. 2022 Jan 27;5(1):e2144727. doi: 10.1001/jamanetworkopen.2021.44727 (PMC8796016; doi:10.1001/jamanetworkopen.2021.44727)
Supplement: Supplement. — eAppendix 1. Description of Sample eTable 1. Details of Data Included and Demographic Characteristics of Participants Included in Main Analysis Compared With Those Who Were Not Due to Missing Data, Pregnancy, or Type 1 Diabetes eAppendix 2. Assessment of Cardiometabolic Outcomes in Adulthood (Age 24 Years) eTable 2. Bivariate Correlations Among Measures of Childhood Socioeconomic Status eAppendix 3. Longitudinal Temperament Profiles Using Repeated Measures of Shyness and Sociability eTable 3. Bivariate Correlations Among Measures Of Shyness and Sociability Across Ages 3 to 5 Years eTable 4. Fit and Quality of Partition Statistics From Longitudinal Clustering Analysis eAppendix 4. Adolescent Moderate to Vigorous Physical Activity (MVPA) eFigure 1. Conceptual Diagram of the Measurement Model of Adolescent Physical Activity eTable 5. Factor Loadings and Fit Indices for the Confirmatory Factor Analysis of Adolescent Physical Activity eFigure 2. Conceptual Figure of the Path Analyses and Structural Equation Models Examining Indirect Effects of Childhood Temperaments on Cardiometabolic Outcomes via Social Occupation Class in Adulthood and Physical Activity in Adolescence eTable 6. Unadjusted and Adjusted Associations Between Early Social and Biological Factors and Child Temperament eTable 7. Unadjusted and Adjusted Associations Between Child Temperament, Adolescent MVPA, and Young Adult Social Occupation Class and Cardiometabolic Indices at Age 24 Years eTable 8. Results From Models Examining the Indirect Effects of Child Temperament on CRP Levels, BMI, Triglyceride Levels, and HDL and LDL Cholesterol Levels at Age 24 Years, via Adolescent Physical Activity eTable 9. Results From Models Examining the Indirect Effects of Child Temperament on Glucose Levels, Insulin Levels, SBP, and DBP at Age 24 Years, via Adolescent Physical Activity eTable 10. Results From Models Examining the Indirect Effects of Child Temperament on CRP Levels, BMI, Triglyceride Levels, and HDL and LDL [file jamanetwopen-e2144727-s001.pdf]

Supplemental Online Content

Tang A, Fox NA, Slopen N. Examination of early childhood temperament of shyness and social avoidance and associations with cardiometabolic health in young adulthood. *JAMA Netw Open.* 2022;5(1):e2144727. doi:10.1001/jamanetworkopen.2021.44727

eAppendix 1. Description of Sample

eTable 1. Details of Data Included and Demographic Characteristics of Participants Included in Main Analysis Compared With Those Who Were Not Due to Missing Data, Pregnancy, or Type 1 Diabetes

eAppendix 2. Assessment of Cardiometabolic Outcomes in Adulthood (Age 24 Years)

eTable 2. Bivariate Correlations Among Measures of Childhood Socioeconomic Status

eAppendix 3. Longitudinal Temperament Profiles Using Repeated Measures of Shyness and Sociability

eTable 3. Bivariate Correlations Among Measures Of Shyness and Sociability Across Ages 3 to 5 Years

eTable 4. Fit and Quality of Partition Statistics From Longitudinal Clustering Analysis

eAppendix 4. Adolescent Moderate to Vigorous Physical Activity (MVPA)

eFigure 1. Conceptual Diagram of the Measurement Model of Adolescent Physical Activity

eTable 5. Factor Loadings and Fit Indices for the Confirmatory Factor Analysis of Adolescent Physical Activity

eFigure 2. Conceptual Figure of the Path Analyses and Structural Equation Models Examining Indirect Effects of Childhood Temperaments on Cardiometabolic Outcomes via Social Occupation Class in Adulthood and Physical Activity in Adolescence

eTable 6. Unadjusted and Adjusted Associations Between Early Social and Biological Factors and Child Temperament

eTable 7. Unadjusted and Adjusted Associations Between Child Temperament, Adolescent MVPA, and Young Adult Social Occupation Class and Cardiometabolic Indices at Age 24 Years

eTable 8. Results From Models Examining the Indirect Effects of Child Temperament on CRP Levels, BMI, Triglyceride Levels, and HDL and LDL Cholesterol Levels at Age 24 Years, via Adolescent Physical Activity

eTable 9. Results From Models Examining the Indirect Effects of Child Temperament on Glucose Levels, Insulin Levels, SBP, and DBP at Age 24 Years, via Adolescent Physical Activity

eTable 10. Results From Models Examining the Indirect Effects of Child Temperament on CRP Levels, BMI, Triglyceride Levels, and HDL and LDL Cholesterol Levels at Age 24 Years, via Adult Social Occupation Class

eTable 11. Results From Models Examining the Indirect Effects of Child Temperament on Glucose Levels, Insulin Levels, SBP, and DBP at Age 24, via Adult Social Occupation Class

eReferences.

© 2022 Tang A et al. *JAMA Network Open.*

This supplemental material has been provided by the authors to give readers additional information about their work.

© 2022 Tang A et al. *JAMA Network Open.*

eAppendix. Description of Sample

The Avon Longitudinal Study of Parents and Children is a birth cohort study from the UK designed to investigate the early and on-going developmental risk factors linked to physical and psychosocial health. A summary of participant recruitment and selection was reported in the main text, along with a flow chart in the main text (Figure 2). This study used temperament data collected in childhood (ages 3-6), accelerometer data collected in adolescence (ages 11-15), and cardiometabolic indices collected in the clinic in adulthood (age 24). We provide missing data numbers and solution in the data analysis section of the main text. For more details about participant recruitment, see previously published papers[1-3]. Also, the ALSPAC website contains details of all the data that is available through a fully searchable data dictionary and variable search tool: http://www.bristol.ac.uk/alspac/researchers/our-data/

© 2022 Tang A et al. *JAMA Network Open.*

eTable 1. Details of Data Included and Demographic Characteristics of Participants Included in Main Analysis Compared With Those Who Were Not Due to Missing Data, Pregnancy, or Type 1 Diabetes

Measures	N Invited (Alive + contact info)	N Responded and attended	N Completed and had valid data	N In longitudinal temperament analysis (2+ time points)
Temperament questionnaire				
Age 3	13,988	10,145	10,051	9,119
Age 5	12,128	9531	9420	9121
Age 6	11,280	8698	8614	8444
Accelerometer				
Age 11	11510	6904	6091	5068
Age 13	11351	6141	4775	3776
Age 15	10692	5509	2355	2070
Age 24 clinic visit	9,997	4,026		
BMI			3973	3291
CRP			3028	2503
Cholesterols, insulin, glucose			3267	2707
Blood pressure			3993	3310
Demographics	Analyzed	Not analyzed		
Sex, N female (%)	4898 (51.8%)	2308 (51.4%)	$\chi^2(1)=.18$, $p=.670$	
Ethnicity, N White (%)	8461 (96.0%)	2814 (91.9%)	χ^2 (1)= 81.31, $p<.001$	
Childhood SES factor, *M* (*SD*)	0.01 (.72)	-.27(.73)	$t(12721)=18.47$, $p<.001$	
Birthweight z-score *M (SD)*	-.17 (.95)	-.27 (.99)	$t(13775)=5.46$, $p<.001$	
Maternal depressive symptoms *M (SD)*	5.96 (4.48)	6.61 (5.10)	$t(10994)= -5.52$, $p< .001$	

Note. Number of participants differed in analyses: n for sex= 13,957, n for ethnicity=12,064, n for childhood SES factor=12,723, n for birthweight z-score= 13,777, n for maternal depressive symptoms=10996. BMI= body mass index. CRP= C-reactive protein.

eAppendix 2. Assessment of Cardiometabolic Outcomes in Adulthood (Age 24 Years)

Assessment of anthropometrics, blood pressure, and fasting blood samples were completed in the clinic. Trained nurses measured participants' height and weight, which were used to calculate body mass index. Sitting systolic and diastolic blood pressure (SBP and DBP) readings were taken twice using an Omron M6 upper arm blood pressure/pulse monitor, after participants were asked to sit for two minutes. The average of the two readings were used in analyses.

Participants were asked to fast overnight or for a minimum of 8 hours before their blood draw. Blood samples were immediately spun and frozen at -80º C. Plasma lipid assays for triglycerides, high-density and low-density lipoprotein (HDL, LDL) cholesterols were conducted through a modification of the standard Lipid Research Clinics Protocol using enzymatic reagents for lipid determination. Insulin was measured by an ELISA assay (Mercodia, Uppsala, Sweden) that does not cross-react with proinsulin and plasma glucose. Glucose was measured with an automated analyzer (Roche Diagnostics, Burgess, UK). High-sensitivity CRP was measured by automated particle enhance immunoturbidimetric assay (Roche Diagnostics, Burgess, UK). All assay coefficients of variation were < 5%. CRP, glucose, and insulin showed severely positively skewed distributions; as such, the log-transformed values were used in further analyses.

© 2022 Tang A et al. *JAMA Network Open.*

eTable 2. Bivariate Correlations Among Measures of Childhood Socioeconomic Status

	1	2	3	4	5	6
1. Family weekly income age 2	--					
2. Family weekly income age 3	.80**	--				
3. Mother's highest education level 32 weeks gestation	.19**	.18**	--			
4. Father's highest education level 32 weeks gestation	.23**	.21**	.63**	--		
5. Father's social occupation class 32 weeks gestation	.38**	.37**	.19**	.23**	--	
6. Mother's social occupation class 32 weeks gestation	.35**	.34**	.21**	.29**	.36**	--
Mean	3.39	3.52	2.17	2.39	3.45	3.34
SD	1.24	1.25	1.29	1.44	0.91	0.80
N	7936	7903	6898	6522	8369	7765

Note. ** p <.001.

© 2022 Tang A et al. *JAMA Network Open.*

eAppendix 3. Longitudinal Temperament Profiles Using Repeated Measures of Shyness and Sociability

First, we determined the internal consistency and temporal stability of shyness and sociability scales in this sample. The two scales showed high internal consistency ($\alpha_{shyness}$= .79 to .83; $\alpha_{sociability}$= .63 to .68), as well as high temporal stability across time, $r's_{shyness}$= .57 to .73; $r's_{sociability}$= .46 to .59 (Supplemental Table S2 below).

To examine whether different temperaments exist across childhood, joint trajectories of shyness and sociability from ages 3-6 were estimated using a non-parametric k-means longitudinal clustering method in the R package "kml3d" [4]. K-means is a simple form of unsupervised machine learning that uses an exploratory hill-climbing algorithm to detect clusters of homogenous subgroups within a larger heterogenous population; "kml3d" uses these principles to detect subgroups based on patterns of several repeatedly measured variables simultaneously. In this procedure, each observation is first arbitrarily assigned to a cluster based on the co-evolution of the variables, then optimal clustering is achieved by repeatedly calculating the mean of each cluster/trajectory and reassigning each observation to its nearest means until no further changes occur. The estimations were repeated 5000 times to obtain optimal solutions (1000 times for each of the 2-, 3-, 4-, 5-, 6- cluster solutions). This analysis included 9491 participants, who had temperament data in at least two of the three time points; the missing data point was imputed through linear interpolation.

Selection of the cluster solution was based on theory and fit/quality of partition statistics, including the Calinski-Harabasz, Davies-Bouldin index, BIC, and global average of posterior-probabilities (i.e., the probability that each participant belongs to their assigned trajectory). Supplemental Table S3 shows the fit/quality of partition statistics. The 4-cluster solution was selected, as the subgroups support prior theory about the four different types of temperament (see Figure 1 in main text): Avoidant-shy, Conflicted-shy, Extraverted, and Introverted). This solution also showed relatively good reliability and separation across indices.

© 2022 Tang A et al. *JAMA Network Open.*

eTable 3. Bivariate Correlations Among Measures Of Shyness and Sociability Across Ages 3 to 5 Years

	1	2	3	4	5	6
1. Shyness age 3	--					
2. Shyness age 5	.63**	--				
3. Shyness age 6	.57**	.73**	--			
4. Sociability age 3	-.46**	-.32**	-.29**	--		
5. Sociability age 5	-.26**	-.41**	-.33**	.51**	--	
6. Sociability age 6	-.23**	-.33**	-.39**	.46**	.59**	--
Mean	12.47	11.92	11.78	18.19	18.17	18.24
SD	4.09	3.24	3.17	3.11	2.63	2.645
N	9123	9121	8445	9119	9140	8444

Note. Analyses include those with temperament data collected for at least two of the three time points (*n*=9492).

© 2022 Tang A et al. *JAMA Network Open.*

eTable 4. Fit and Quality of Partition Statistics From Longitudinal Clustering Analysis

	K- cluster solutions				
	2	3	4	5	6
Calinski-Harabasz criterion	4599.56	4845.37	5319.26	5361.60	5368.77
Davies-Bouldin index	-1.42	-1.52	-1.46	-1.50	-1.50
BIC	-140761.38	-137936.57	-136255.42	-135281.76	-134788.58
Global average of posterior-probabilities	.92	.86	.84	.81	.79
% in each group					
group 1	55%	46%	30%	24%	23%
group 2	45%	32%	27%	24%	19%
group 3		22%	24%	21%	17%
group 4			20%	18%	15%
group 5				13%	15%
group 6					10%

Note. Better separation of clusters is suggested by higher values of the Calinski-Harabasz criterion and lower values of the Davies-Bouldin index. Lower values of the BIC suggest better fit of the data. The global average of posterior-probabilities represents the probability that each individual belongs to their assigned clusters, or in other words, how reliable the cluster assignments are. In selecting the cluster solution, we considered a combination of these indices, as well as prior theory about different types of temperament.

© 2022 Tang A et al. *JAMA Network Open.*

eAppendix 4. Adolescent Moderate to Vigorous Physical Activity (MVPA)

Measurement and Processing. At ages 11, 13, and 15, children who attended the research clinics were asked to wear an Actigraph AM7164 2.2 accelerometer (Actigraph LLC, FL, USA) around the waist for a week, during waking hours, except when showering, bathing, or playing water sports. Accelerometry measures of physical activity has been validated in both children and adolescents[5,6]. The accelerometer detects acceleration and deceleration in a vertical plane as a combined function of movement frequency and intensity. Data were recorded as counts, averaged over one-minute periods. The data were downloaded with the Actigraph Reader Interface unite RIU-41A with RIU software (version 2.26B, Actigraph LLC). Data were considered valid and reliable, if the accelerometer had been worn for at least 10 hours a day for at least three days of the week, after deletion of missing data (i.e., periods with ≥10 minutes of successive zeros was regarded as non-wear time and were deleted)[6].

MVPA is defined as any activity that is equivalent to the physiological stress of brisk walking for a person of average fitness[7]. Activities that pass the threshold of moderate intensity in vigor include any aerobic activity that causes increased heart rate and potential sweating (e.g., running, cycling, dancing). Other activities, including jogging, hill walking and racket sports, are also considered vigorous. In the Actigraph recordings, time spent in MVPA is identified as minutes with > 3600 accelerometer counts. The threshold of 3600 counts per minute was determined from a calibration study conducted in a subsample of 246 ALSPAC children[6]. This threshold is four times greater than the resting metabolic rate, approximates activity from brisk walking, and has predictive validity as it is associated with reduced fat mass in these children[7]. Weekly MVPA was calculated as the average minutes with >3600 counts per valid day; Log-transformation was used to reduce positive skewness in weekly MVPA values and used in further analyses. Weekly MVPA was moderately correlated across adolescence (*r's*=.39 to .46, *p's* <.001).

Confirmatory factor analysis of adolescent physical activity. To maximize the available data and provide a robust and valid measure of physical activity to use in analyses, we used a confirmatory factor analysis (CFA). Adolescent physical activity was measured as a latent construct using weekly MVPA measured at ages 11, 13, and 15 as indicators (Supplemental Figure S1). CFA of the measurement model revealed good fit and all loadings were > .40 (Supplemental Table S4).

© 2022 Tang A et al. *JAMA Network Open.*

eFigure 1. Conceptual Diagram of the Measurement Model of Adolescent Physical Activity

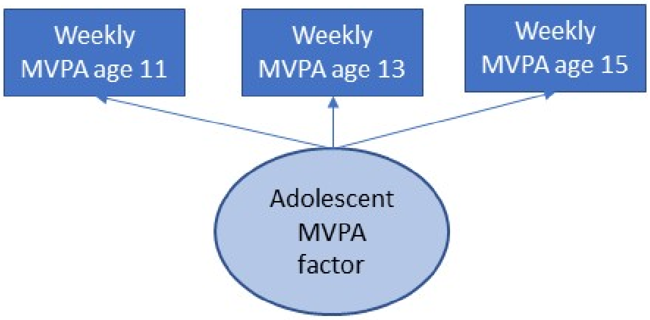

Note. MVPA= moderate-vigorous physical activity.

© 2022 Tang A et al. *JAMA Network Open.*

eTable 5. Factor Loadings and Fit Indices for the Confirmatory Factor Analysis of Adolescent Physical Activity

	β	b	SE	Z	P
Log weekly MVPA age 11	.64	.08	.003	29.06	< .001
Log weekly MVPA age 13	.74	.11	.004	29.89	< .001
Log weekly MVPA age 15	.63	.09	.004	23.67	< .001
Fit indices					
CFI	1.00				
RMSEA	0.00				
SRMR	0.00				
$\chi 2$ goodness of fit	df=3, 1283.02, p< .001				

Note. MVPA= moderate-vigorous physical activity.
N=5597 in confirmatory factor analysis, including participants with at least one data point of physical activity.

© 2022 Tang A et al. *JAMA Network Open.*

eFigure 2. Conceptual Figure of the Path Analyses and Structural Equation Models Examining Indirect Effects of Childhood Temperaments on Cardiometabolic Outcomes via Social Occupation Class in Adulthood and Physical Activity in Adolescence

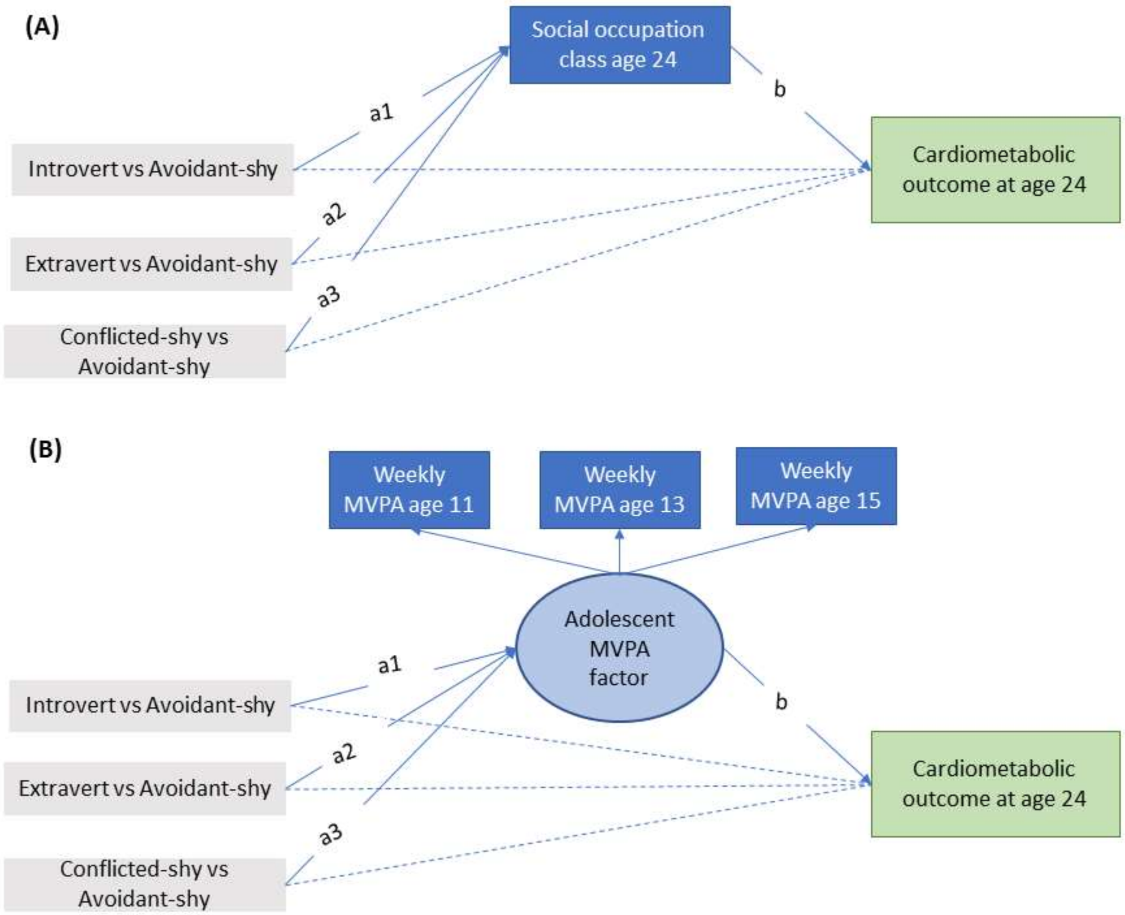

Note. MVPA= moderate-vigorous physical activity. Temperament was coded as 3 dummy variables, with *Avoidant-shyness* as the reference group (i.e., *Introvert* vs. *Avoidant-shy, Extravert* vs. *Avoidant-shy*, *Conflicted-shy* vs. *Avoidant-shy*).
Three sets of indirect effects were tested in follow-up tests with Monte Carlo simulations that derive 95% confidence intervals: a1*b, a2*b, a3*b.

Dotted lines represent paths that are not statistically significant, solid lines represent paths that are statistically significant. Please see Supplemental Tables S8-10 for the exact estimates.
For simplicity, covariates and regression paths accounting for child's sex, ethnicity, childhood SES, maternal depressive symptoms, birthweight, and BMI at age 24 are not shown. Residual covariances among predictors were included but are also not shown.

© 2022 Tang A et al. *JAMA Network Open.*

eTable 6. Unadjusted and Adjusted Associations Between Early Social and Biological Factors and Child Temperament

	Introvert vs Avoidant-shy			Extravert vs Avoidant-shy			Conflicted-shy vs Avoidant-shy		
	OR	95% CI	p	OR	95% CI	p	OR	95% CI	p
Unadjusted estimates									
Childhood SES	1.15	(1.06, 1.25)	.001	1.24	(1.14, 1.35)	.000	1.13	(1.04, 1.23)	.005
Maternal depressive symptoms	.97	(.96, .99)	<.001	1.00	(.98, 1.01)	.473	1.00	(.98, 1.01)	.451
Birthweight z-score	1.04	(.98, 1.11)	.198	.96	(.90, 1.03)	.250	.99	(.93, 1.06)	.737
Adjusted estimates									
Childhood SES	1.13	(1.04, 1.23)	.001	1.25	(1.14, 1.36)	.000	1.13	(1.04, 1.23)	.002
Maternal depressive symptoms	.98	(.96, .99)	<.001	1.00	(.99, 1.01)	.883	1.00	(.98, 1.01)	.685
Birthweight z-score	1.03	(.97, 1.10)	.282	.95	(.89, 1.01)	.110	.98	(.92, 1.04)	.479

Note. SES= socioeconomic status.
N in models= 9491.
Adjusted estimates analyses are based on a model including childhood SES, maternal depressive symptoms, birthweight z-score, sex, and child ethnicity.

© 2022 Tang A et al. *JAMA Network Open.*

eTable 7. Unadjusted and Adjusted Associations Between Child Temperament, Adolescent MVPA, and Young Adult Social Occupation Class and Cardiometabolic Indices at Age 24 Years

	Introvert vs Avoidant-shy			Extravert vs Avoidant-shy			Conflicted-shy vs Avoidant-shy		
Outcomes	β	95% CI	p	β	95% CI	p	β	95% CI	p
Unadjusted models									
Adolescent MVPA factor	.10	(.05, .14)	<.001	.11	(.07, .16)	<.001	.05	(.00, .09)	.041
Adult social occupation class	-.07	(-.12, -.02)	.007	-.08	(-.13, -.04)	.001	-.08	(-.13, -.03)	.001
CRP	-.03	(-.08, .02)	.255	.02	(-.03, .07)	.795	-.03	(-.08, .03)	.320
BMI	.07	(.02, .12)	.003	.10	(.06, .15)	.000	.05	(.01, .10)	.020
Triglycerides	.02	(-.03, .07)	.478	.00	(-.05, .05)	.961	-.02	(-.07, .03)	.416
LDL	.02	(-.03, .07)	.424	.02	(-.03, .07)	.524	-.01	(-.06, .04)	.620
HDL	-.04	(-.09, .02)	.178	.02	(-.03, .07)	.515	.02	(-.03, .07)	.404
Glucose	.02	(-.03, .08)	.349	.01	(-.04, .06)	.668	.02	(-.03, .07)	.379
Insulin	-.01	(-.06, .04)	.811	-.01	(-.06, .04)	.740	-.01	(-.06, .04)	.733
SBP	.01	(-.03, .06)	.563	.01	(-.04, .05)	.785	.00	(-.05, .04)	.868
DBP	-.02	(-.06, .03)	.483	.02	(-.03, .06)	.443	.00	(-.05, .04)	.972
Adjusted models									
Adolescent MVPA factor	.10	(.06, .14)	<.001	.14	(.10, .18)	<.001	.09	(.05, .13)	<.001
Adult social occupation class	-.06	(-.11, -.01)	.015	-.07	(-.12, -.02)	.004	-.06	(-.11, -.02)	.007
CRP	-.05	(-.10, .00)	.055	-.02	(-.07, .03)	.472	-.05	(-.10, .00)	.036
BMI	.08	(.03, .12)	.002	.11	(.07, .16)	<.001	.06	(.01, .10)	.009
Triglycerides	-.01	(-.06, .04)	.666	-.03	(-.08, .02)	.250	-.03	(-.08, .01)	.169
LDL	.00	(-.05, .05)	.969	-.01	(-.06, .04)	.723	-.03	(-.07, .02)	.288
HDL	-.01	(-.05, .04)	.789	.03	(-.02, .08)	.187	.02	(-.02, .07)	.345
Glucose	.01	(-.04, .06)	.728	.01	(-.04, .06)	.790	.03	(-.02, .07)	.315
Insulin	-.04	(-.09, .00)	.073	-.06	(-.10, -.01)	.012	-.04	(-.08, .01)	.092
SBP	-.01	(-.05, .02)	.470	.00	(-.04, .04)	.920	.01	(-.03, .04)	.647
DBP	-.04	(-.08, .00)	.056	-.02	(-.06, .03)	.439	-.01	(-.06, .03)	.508

Note. MVPA= moderate-vigorous physical activity. CRP= C-reactive protein. BMI= Body mass index. LDL= Low-density lipoprotein. HDL= High-density lipoprotein. SBP= Systolic blood pressure. DBP= diastolic blood pressure.

N in models= 9457, excluding those who reported type-1 diabetes and pregnant females.

Adjusted estimates for adult outcomes are based on models including covariates for sex, child ethnicity, childhood SES, maternal depressive symptoms, birthweight z-score, and BMI at age 24. Adjusted estimates for adolescent MVPA included all covariates except BMI at age 24. The analysis for BMI does not include itself as a covariate.

© 2022 Tang A et al. *JAMA Network Open.*

eTable 8. Results From Models Examining the Indirect Effects of Child Temperament on CRP Levels, BMI, Triglyceride Levels, and HDL and LDL Cholesterol Levels at Age 24 Years, via Adolescent Physical Activity

	CRP			BMI			Triglycerides			LDL			HDL		
	β	b (95% CI)	p	β	b (95% CI)	p	β	b (95% CI)	p	β	b (95% CI)	p	β	b (95% CI)	p
Effect on adolescent MVPA factor															
Introvert vs Avoidant-shy	.10	.25 (.14, .35)	.000	.10	.25 (.14, .35)	.000	.10	.25 (.14, .35)	.000	.10	.25 (.14, .35)	.000	.10	.25 (.14, .35)	.000
Extravert vs Avoidant-shy	.14	.36 (.25, .47)	.000	.14	.36 (.25, .47)	.000	.14	.36 (.25, .47)	.000	.14	.36 (.25, .47)	.000	.14	.36 (.25, .47)	.000
Conflict-shy vs Avoidant-shy	.09	.23 (.12, .34)	.000	.09	.23 (.12, .34)	.000	.09	.23 (.12, .34)	.000	.09	.23 (.12, .34)	.000	.09	.23 (.12, .34)	.000
Sex (female)	-.45	-1.01 (-1.09, -.93)	.000	-.45	-1.01 (-1.09, -.93)	.000	-.45	-1.01 (-1.09, -.93)	.000	-.45	-1.01 (-1.09, -.93)	.000	-.45	-1.01 (-1.09, -.93)	.000
Childhood SES	-.09	-.14 (-.19, -.09)	.000	-.09	-.14 (-.19, -.09)	.000	-.09	-.14 (-.19, -.09)	.000	-.09	-.14 (-.19, -.09)	.000	-.09	-.14 (-.19, -.09)	.000
Maternal depressive symptoms	-.03	-.01 (-.02, .00)	.137	-.03	-.01 (-.02, .00)	.136	-.03	-.01 (-.02, .00)	.135	-.03	-.01 (-.02, .00)	.133	-.03	-.01 (-.02, .00)	.130
Child ethnicity	-.03	-.18 (-.37, .02)	.079	-.03	-.18 (-.37, .02)	.079	-.03	-.18 (-.38, .02)	.078	-.03	-.18 (-.38, .02)	.074	-.03	-.18 (-.38, .02)	.076
Birthweight z-score	.01	.01 (-.03, .05)	.682	.01	.01 (-.03, .05)	.681	.01	.01 (-.03, .05)	.681	.01	.01 (-.03, .05)	.684	.01	.01 (-.03, .05)	.679
Effect on outcome age24															
Adolescent MVPA factor	.01	.00 (-.01, .01)	.793	-.07	-.32 (-.56, -.07)	.012	-.04	-1.56 (-3.87, .76)	.189	-.07	-1.69 (-3.32, -.06)	.042	.07	.95 (.12, 1.78)	.025
Introvert vs Avoidant-shy	-.05	-.02 (-.03, .00)	.053	.08	.84 (.36, 1.33)	.001	-.01	-.58 (-5.09, 3.94)	.803	.01	.38 (-2.80, 3.57)	.813	-.01	-.47 (-2.09, 1.15)	.569
Extravert vs Avoidant-shy	-.02	-.01 (-.02, .01)	.449	.12	1.35 (.84, 1.85)	.000	-.02	-2.18 (-6.86, 2.51)	.362	.00	.01 (-3.29, 3.32)	.994	.02	.77 (-.91, 2.45)	.368
Conflict-shy vs Avoidant-shy	-.05	-.02 (-.03, .00)	.034	.06	.73 (.23, 1.22)	.004	-.03	-2.93 (-7.62, 1.75)	.220	-.02	-1.37 (-4.68, 1.93)	.416	.02	.57 (-1.11, 2.25)	.506
Sex (female)	.18	.05 (.04, .06)	.000	-.04	-.41 (-.82, .00)	.050	-.17	-14.34 (-18.14, -1.54)	.000	-.05	-2.89 (-5.57, -.21)	.034	.33	1.85 (9.49, 12.22)	.000
Childhood SES	-.03	-.01 (-.01, .00)	.127	-.15	-1.04 (-1.28, -.79)	.000	-.02	-1.36 (-3.63, .92)	.242	-.05	-2.05 (-3.65, -.44)	.012	.14	3.11 (2.29, 3.92)	.000
Maternal depressive symptoms	.04	.00 (.00, .00)	.043	.02	.02 (-.02, .06)	.307	.01	.11 (-.26, .47)	.566	-.05	-.33 (-.58, -.07)	.012	-.01	-.02 (-.15, .11)	.777
Child ethnicity	-.01	-.01 (-.04, .02)	.610	-.01	-.25 (-1.12, .62)	.578	-.05	-1.11 (-18.55, -1.66)	.019	-.01	-1.67 (-7.63, 4.29)	.583	.06	4.59 (1.57, 7.62)	.003
Birthweight z-score	-.06	-.01 (-.01, .00)	.004	.09	.46 (.28, .64)	.000	-.07	-3.13 (-4.79, -1.47)	.000	-.05	-1.47 (-2.65, -.30)	.014	.04	.65 (.05, 1.25)	.033
BMI age 24	.31	.01 (.01, .01)	.000				.31	2.74 (2.41, 3.07)	.000	.25	1.50 (1.26, 1.73)	.000	-.30	-.99 (-1.11, -.87)	.000

Note. *N*= 9457 in analyses, excluding those who reported type-1 diabetes and pregnant females. All analyses adjusted for sex, child ethnicity, childhood SES, maternal depressive symptoms, birthweight z-score, and BMI at age 24. The analysis for BMI does not include itself as a covariate.

© 2022 Tang A et al. *JAMA Network Open.*

eTable 9. Results From Models Examining the Indirect Effects of Child Temperament on Glucose Levels, Insulin Levels, SBP, and DBP at Age 24 Years, via Adolescent Physical Activity

	Glucose			Insulin			SBP			DBP		
	β	b (95% CI)	p	β	b (95% CI)	p	β	b (95% CI)	p	β	b (95% CI)	p
Effect on adolescent MVPA factor												
Introvert vs Avoidant-shy	.10	.25 (.14, .35)	.000	.10	.25 (.14, .35)	.000	.10	.25 (.14, .35)	.000	.10	.25 (.14, .35)	.000
Extravert vs Avoidant-shy	.14	.36 (.25, .47)	.000	.14	.36 (.25, .47)	.000	.14	.36 (.25, .47)	.000	.14	.36 (.25, .47)	.000
Conflict-shy vs Avoidant-shy	.09	.23 (.12, .34)	.000	.09	.23 (.12, .34)	.000	.09	.23 (.12, .34)	.000	.09	.23 (.12, .34)	.000
Sex (female)	-.45	-1.01 (-1.09, -.93)	.000	-.45	-1.01 (-1.09, -.93)	.000	-.45	-1.01 (-1.09, -.93)	.000	-.45	-1.01 (-1.09, -.93)	.000
Childhood SES	-.09	-.14 (-.19, -.09)	.000	-.09	-.14 (-.19, -.09)	.000	-.09	-.14 (-.19, -.09)	.000	-.09	-.14 (-.19, -.09)	.000
Maternal depressive symptoms	-.03	-.01 (-.02, .00)	.137	-.03	-.01 (-.02, .00)	.135	-.03	-.01 (-.02, .00)	.137	-.03	-.01 (-.02, .00)	.140
Child ethnicity	-.03	-.18 (-.38, .02)	.078	-.03	-.18 (-.38, .02)	.075	-.03	-.18 (-.37, .02)	.078	-.03	-.18 (-.38, .02)	.078
Birthweight z-score	.01	.01 (-.03, .05)	.674	.01	.01 (-.03, .05)	.680	.01	.01 (-.03, .05)	.687	.01	.01 (-.03, .05)	.717
Effect on outcome age24												
Adolescent MVPA factor	.00	.00 (-.03, .03)	.934	-.07	-.57 (-1.04, -.10)	.016	-.01	-.11 (-.59, .37)	.656	-.08	-.59 (-.97, -.21)	.002
Introvert vs Avoidant-shy	.01	.01 (-.05, .08)	.723	-.03	-.69 (-1.61, .24)	.146	-.01	-.32 (-1.25, .62)	.507	-.03	-.57 (-1.31, .17)	.128
Extravert vs Avoidant-shy	.01	.01 (-.06, .08)	.783	-.05	-1.01 (-1.97, -.05)	.039	.00	-.01 (-.98, .96)	.981	-.01	-.10 (-.86, .67)	.807
Conflict-shy vs Avoidant-shy	.03	.04 (-.03, .10)	.313	-.03	-.68 (-1.64, .28)	.165	.01	.25 (-.72, 1.21)	.615	-.01	-.13 (-.89, .64)	.743
Sex (female)	-.22	-.27 (-.32, -.21)	.000	.01	.17 (-.61, .94)	.669	-.50	-11.55 (-12.35, -1.76)	.000	-.12	-1.86 (-2.49, -1.23)	.000
Childhood SES	-.01	-.01 (-.04, .03)	.754	-.05	-.70 (-1.16, -.23)	.003	.02	.28 (-.20, .75)	.256	-.01	-.08 (-.46, .30)	.682
Maternal depressive symptoms	.00	.00 (-.01, .01)	.884	.01	.02 (-.06, .09)	.653	-.02	-.06 (-.13, .02)	.137	-.01	-.02 (-.08, .04)	.485
Child ethnicity	-.04	-.12 (-.27, .03)	.115	.00	.06 (-1.67, 1.80)	.943	-.03	-1.59 (-3.28, .11)	.066	-.01	-.27 (-1.61, 1.08)	.699
Birthweight z-score	-.05	-.03 (-.06, -.01)	.013	-.06	-.63 (-.96, -.29)	.000	-.05	-.60 (-.95, -.25)	.001	-.06	-.49 (-.76, -.21)	.001
BMI age 24	.13	.02 (.01, .02)	.000	.44	.84 (.77, .91)	.000	.31	.74 (.67, .80)	.000	.37	.60 (.54, .65)	.000

Note. *N*= 9457 in analyses, excluding those who reported type-1 diabetes and pregnant females. All analyses adjusted for sex, child ethnicity, childhood SES, maternal depressive symptoms, birthweight z-score, and BMI at age 24.

© 2022 Tang A et al. *JAMA Network Open.*

eTable 10. Results From Models Examining the Indirect Effects of Child Temperament on CRP Levels, BMI, Triglyceride Levels, and HDL and LDL Cholesterol Levels at Age 24 Years, via Adult Social Occupation Class

	CRP			BMI			Triglycerides			LDL			HDL		
	β	b (95% CI)	p	β	b (95% CI)	p	β	b (95% CI)	p	β	b (95% CI)	p	β	b (95% CI)	p
Effect on adult social occupation class															
Introvert vs Avoidant-shy	-.06	-.21 (-.38, -.04)	.015	-.06	-.21 (-.38, -.04)	.016	-.06	-.21 (-.38, -.04)	.016	-.06	-.21 (-.38, -.04)	.015	-.06	-.21 (-.38, -.04)	.016
Extravert vs Avoidant-shy	-.07	-.26 (-.43, -.08)	.004	-.07	-.26 (-.43, -.08)	.004	-.07	-.26 (-.43, -.08)	.004	-.07	-.26 (-.43, -.08)	.004	-.07	-.26 (-.43, -.08)	.004
Conflict-shy vs Avoidant-shy	-.06	-.24 (-.41, -.07)	.007	-.06	-.24 (-.41, -.06)	.007	-.06	-.24 (-.41, -.06)	.007	-.06	-.24 (-.41, -.07)	.007	-.06	-.24 (-.41, -.06)	.007
Sex (female)	-.06	-.21 (-.33, -.09)	.001	-.06	-.21 (-.33, -.09)	.001	-.06	-.21 (-.33, -.09)	.001	-.07	-.21 (-.33, -.09)	.001	-.06	-.21 (-.33, -.09)	.001
Childhood SES	-.19	-.43 (-.52, -.34)	.000	-.19	-.43 (-.52, -.34)	.000	-.19	-.43 (-.52, -.34)	.000	-.19	-.43 (-.52, -.34)	.000	-.19	-.43 (-.52, -.34)	.000
Maternal depressive symptoms	.01	.01 (-.01, .02)	.493	.01	.01 (-.01, .02)	.495	.01	.01 (-.01, .02)	.496	.01	.01 (-.01, .02)	.475	.01	.01 (-.01, .02)	.484
Child ethnicity	.01	.06 (-.26, .38)	.708	.01	.05 (-.27, .37)	.745	.01	.06 (-.26, .38)	.712	.01	.06 (-.27, .38)	.734	.01	.06 (-.26, .38)	.704
Birthweight z-score	-.04	-.06 (-.13, .00)	.048	-.04	-.06 (-.13, .00)	.046	-.04	-.06 (-.13, .00)	.049	-.04	-.06 (-.13, .00)	.051	-.04	-.06 (-.13, .00)	.048
Effect on outcome age24															
Adult social occupation class	.02	.00 (.00, .01)	.522	.07	.21 (.08, .35)	.002	-.01	-.14 (-1.42, 1.13)	.826	-.03	-.63 (-1.50, .25)	.160	-.02	-.16 (-.61, .29)	.477
Introvert vs Avoidant-shy	-.05	-.02 (-.03, .00)	.061	.08	.82 (.34, 1.29)	.001	-.01	-1.02 (-5.50, 3.47)	.657	.00	-.20 (-3.36, 2.97)	.903	-.01	-.25 (-1.86, 1.36)	.759
Extravert vs Avoidant-shy	-.02	-.01 (-.02, .01)	.502	.12	1.30 (.80, 1.79)	.000	-.03	-2.77 (-7.39, 1.85)	.240	-.01	-.76 (-4.02, 2.50)	.648	.03	1.07 (-.59, 2.73)	.206
Conflict-shy vs Avoidant-shy	-.05	-.02 (-.03, .00)	.039	.06	.70 (.20, 1.20)	.006	-.03	-3.34 (-8.00, 1.32)	.161	-.03	-1.90 (-5.19, 1.38)	.256	.02	.77 (-.90, 2.45)	.364
Sex (female)	.18	.05 (.04, .06)	.000	-.01	-.06 (-.40, .28)	.726	-.15	-12.90 (-16.02, -9.79)	.000	-.02	-1.43 (-3.63, .77)	.203	.30	9.92 (8.80, 11.04)	.000
Childhood SES	-.03	-.01 (-.01, .00)	.165	-.14	-.92 (-1.17, -.67)	.000	-.02	-1.21 (-3.53, 1.10)	.304	-.05	-2.09 (-3.72, -.45)	.012	.13	2.92 (2.09, 3.75)	.000
Maternal depressive symptoms	.04	.00 (.00, .00)	.044	.02	.02 (-.02, .06)	.260	.01	.12 (-.24, .48)	.512	-.05	-.31 (-.57, -.06)	.017	-.01	-.03 (-.16, .10)	.682
Child ethnicity	-.01	-.01 (-.04, .02)	.586	-.01	-.18 (-1.05, .69)	.682	-.04	-9.60 (-18.03, -1.18)	.025	-.01	-1.04 (-6.98, 4.90)	.731	.05	4.35 (1.33, 7.36)	.005
Birthweight z-score	-.06	-.01 (-.01, .00)	.005	.09	.47 (.29, .65)	.000	-.07	-3.16 (-4.82, -1.49)	.000	-.05	-1.54 (-2.72, -.37)	.010	.04	.65 (.05, 1.25)	.034
BMI age 24	.31	.01 (.01, .01)	.000				.31	2.76 (2.43, 3.09)	.000	.25	1.52 (1.29, 1.75)	.000	-.30	-1.00 (-1.12, -.88)	.000

Note. *N*= 9457 in analyses, excluding those who reported type-1 diabetes and pregnant females. All analyses adjusted for sex, child ethnicity, childhood SES, maternal depressive symptoms, birthweight z-score, and BMI at age 24. The analysis for BMI does not include itself as a covariate.

© 2022 Tang A et al. *JAMA Network Open.*

eTable 11. Results From Models Examining the Indirect Effects of Child Temperament on Glucose Levels, Insulin Levels, SBP, and DBP at Age 24, via Adult Social Occupation Class

	Glucose			Insulin			SBP			DBP		
	β	b (95% CI)	p	β	b (95% CI)	p	β	b (95% CI)	p	β	b (95% CI)	p
Effect on adult social occupation class												
Introvert vs Avoidant-shy	-.06	-.21 (-.38, -.04)	.016	-.06	-.21 (-.38, -.04)	.016	-.06	-.21 (-.38, -.04)	.016	-.06	-.21 (-.38, -.04)	.015
Extravert vs Avoidant-shy	-.07	-.26 (-.43, -.08)	.004	-.07	-.26 (-.43, -.08)	.004	-.07	-.26 (-.43, -.08)	.004	-.07	-.26 (-.43, -.08)	.004
Conflict-shy vs Avoidant-shy	-.06	-.24 (-.41, -.06)	.008	-.06	-.24 (-.41, -.06)	.007	-.06	-.24 (-.41, -.07)	.007	-.06	-.24 (-.41, -.07)	.007
Sex (female)	-.06	-.21 (-.33, -.09)	.001	-.06	-.21 (-.33, -.09)	.001	-.06	-.21 (-.33, -.09)	.001	-.06	-.21 (-.33, -.09)	.001
Childhood SES	-.19	-.43 (-.52, -.34)	.000	-.19	-.43 (-.52, -.34)	.000	-.19	-.43 (-.52, -.34)	.000	-.19	-.43 (-.52, -.34)	.000
Maternal depressive symptoms	.01	.01 (-.01, .02)	.493	.01	.01 (-.01, .02)	.488	.01	.01 (-.01, .02)	.490	.01	.01 (-.01, .02)	.492
Child ethnicity	.01	.06 (-.26, .38)	.712	.01	.06 (-.26, .38)	.711	.01	.07 (-.25, .39)	.660	.01	.07 (-.26, .39)	.687
Birthweight z-score	-.04	-.06 (-.13, .00)	.047	-.04	-.06 (-.13, .00)	.049	-.04	-.06 (-.13, .00)	.050	-.04	-.06 (-.13, .00)	.049
Effect on outcome age24												
Adult social occupation class	.01	.01 (-.02, .03)	.689	.01	.03 (-.26, .32)	.845	-.04	-.25 (-.51, .00)	.054	-.02	-.11 (-.31, .09)	.293
Introvert vs Avoidant-shy	.01	.01 (-.05, .08)	.707	-.04	-.83 (-1.75, .09)	.076	-.02	-.40 (-1.33, .53)	.399	-.04	-.74 (-1.47, .00)	.049
Extravert vs Avoidant-shy	.01	.01 (-.06, .08)	.763	-.06	-1.21 (-2.15, -.26)	.013	-.01	-.12 (-1.08, .84)	.803	-.02	-.33 (-1.09, .43)	.393
Conflict-shy vs Avoidant-shy	.03	.04 (-.03, .10)	.303	-.04	-.81 (-1.77, .14)	.095	.01	.17 (-.79, 1.13)	.726	-.02	-.28 (-1.04, .48)	.472
Sex (female)	-.22	-.26 (-.31, -.22)	.000	.04	.71 (.07, 1.35)	.029	-.50	-11.51 (-12.16, -1.85)	.000	-.08	-1.32 (-1.84, -.80)	.000
Childhood SES	.00	.00 (-.04, .03)	.855	-.05	-.62 (-1.09, -.14)	.011	.01	.19 (-.29, .68)	.432	.00	-.05 (-.43, .34)	.813
Maternal depressive symptoms	.00	.00 (-.01, .01)	.882	.01	.02 (-.05, .10)	.555	-.02	-.06 (-.13, .02)	.145	-.01	-.02 (-.08, .04)	.588
Child ethnicity	-.04	-.12 (-.27, .03)	.112	.01	.22 (-1.51, 1.95)	.805	-.03	-1.53 (-3.22, .16)	.075	.00	-.10 (-1.44, 1.24)	.884
Birthweight z-score	-.05	-.03 (-.06, -.01)	.015	-.06	-.63 (-.97, -.29)	.000	-.05	-.62 (-.97, -.27)	.000	-.06	-.50 (-.78, -.23)	.000
BMI age 24	.13	.02 (.01, .02)	.000	.44	.85 (.78, .91)	.000	.32	.74 (.68, .81)	.000	.37	.61 (.55, .66)	.000

Note. *N*= 9457 in analyses, excluding those who reported type-1 diabetes and pregnant females. All analyses adjusted for sex, child ethnicity, childhood SES, maternal depressive symptoms, birthweight z-score, and BMI at age 24.

© 2022 Tang A et al. *JAMA Network Open.*

References

1. Northstone K, Lewcock M, Groom A, et al. The Avon Longitudinal Study of Parents and Children (ALSPAC): an update on the enrolled sample of index children in 2019. *Wellcome open research* 2019;4
2. Boyd A, Golding J, Macleod J, et al. Cohort profile: the 'children of the 90s'—the index offspring of the Avon Longitudinal Study of Parents and Children. *International journal of epidemiology* 2013;42(1):111-27.
3. Fraser A, Macdonald-Wallis C, Tilling K, et al. Cohort profile: the Avon Longitudinal Study of Parents and Children: ALSPAC mothers cohort. *International journal of epidemiology* 2013;42(1):97-110.
4. Genolini C, Alacoque X, Sentenac M, et al. kml and kml3d: R packages to cluster longitudinal data. *Journal of Statistical Software* 2015;65(4):1-34.
5. Trost SG, Loprinzi PD, Moore R, et al. Comparison of accelerometer cut points for predicting activity intensity in youth. *Med Sci Sports Exerc* 2011;43(7):1360-68.
6. Mattocks C, Leary S, Ness A, et al. Calibration of an accelerometer during free-living activities in children. *International Journal of Pediatric Obesity* 2007;2(4):218-26.
7. Riddoch CJ, Leary SD, Ness AR, et al. Prospective associations between objective measures of physical activity and fat mass in 12-14 year old children: the Avon Longitudinal Study of Parents and Children (ALSPAC). *Bmj* 2009;339

© 2022 Tang A et al. *JAMA Network Open.*
